# Effects of the Limestone Particle Size on the Sulfation Reactivity at Low SO_2_ Concentrations Using a LC-TGA

**DOI:** 10.3390/ma12091496

**Published:** 2019-05-08

**Authors:** Runxia Cai, Yiqun Huang, Yiran Li, Yuxin Wu, Hai Zhang, Man Zhang, Hairui Yang, Junfu Lyu

**Affiliations:** 1 Key Laboratory for Thermal Science and Power Engineering of Ministry of Education, Department of Energy and Power Engineering, Tsinghua University, Haidian District, Beijing 100084, China; cairx14@mails.tsinghua.edu.cn (R.C.); huangyq1993@163.com (Y.H.); li-yr15@mails.tsinghua.edu.cn (Y.L.); wuyx09@mail.tsinghua.edu.cn (Y.W.); haizhang@mail.tsinghua.edu.cn (H.Z.); yhr@mail.tsinghua.edu.cn (H.Y.); lvjf@mail.tsinghua.edu.cn (J.L.); 2 Tsinghua University-University of Waterloo Joint Research Center for Micro/Nano Energy and Environment Technology, Tsinghua University, Haidian District, Beijing 100084, China

**Keywords:** Limestone, particle size, sulfation, TGA, model

## Abstract

Limestone particle size has a crucial influence on SO_2_ capture efficiency, however there are few studies on the sulfation reactivity, which covers a broad range of particle sizes at low SO_2_ concentrations. In this paper, a large-capacity thermogravimetric analyzer (LC-TGA) was developed to obtain the sulfur removal reaction rate under a wide range of particle sizes (3 μm–600 μm) and SO_2_ concentrations (250 ppm–2000 ppm), and then compared with the results of a traditional fixed bed reactor and a commercial TGA. The experimental results showed that the LC-TGA can well eliminate the external mass transfer and obtain a better measurement performance. Both the final conversion and the reaction rate reduced with the decreasing of SO_2_ concentration, but ultrafine limestone particles still showed the good sulfation reactivity even at 250 ppm SO_2_. An empirical sulfation model was established based on the experimental results, which can well predict the sulfation process of different limestone particle sizes at low SO_2_ concentrations. The model parameters have a strong negative correlation against the particle size, and the fit of the reaction order of SO_2_ was found to be about 0.6. The model form is very simple to incorporate it into available fluidized bed combustion models to predict SO_2_ emission.

## 1. Introduction

Limestone is widely used for SO_2_ capture in circulating fluidized bed (CFB) boilers for its low price and availability [1,2]. Under air combustion conditions, limestone involves first calcination to the porous CaO, and then the reaction with sulfur containing gas to CaSO_4_ [3]. As the molar volume of CaSO_4_ is about three times larger than that of CaO [4], pore filling or pore plugging on the surface of a CaO particle will block the further reaction in the inner core. Thus, one of the main drawbacks of desulfurization by limestone is the low utilization rate of calcium [5].

Many factors will influence the sulfation process and the maximum calcium utilization rate, such as temperature, limestone properties, steam, particles size, SO_2_ concentration and so on [6]. SO_2_ capture is strongly affected by the temperature. High temperature will cause the sintering of sorbent particles and thermodynamic instability of CaSO_4_ under reducing conditions, and low temperature will reduce the calcination rate and inhibit the pore development, thus the maximum sulfur capture efficiency in atmospheric fluidized beds is usually achieved at 850 °C or a little lower [7,8,9,10]. Limestones vary greatly in properties, and the geological properties also have strong influence on the reactivity of CaO. The consensus view is that older limestones tend to be more compact and less reactive than younger limestones [6]. Steam can also affect calcination, sintering and sulfation reactions of the limestone, and a small amount of water vapor may have positive effect on the calcium conversion rate [11].

Limestone particle size also has a crucial influence on SO_2_ capture efficiency. Small pores result in a high reaction rate but will be easily plugged during the sulfation process [12], so only the superficial surface layer participates in the reaction [13]. Since this superficial area of a sorbent particle increases directly with the decreasing of particle size, the reaction rate of smaller limestone is much higher than that of larger ones. Due to the formation of the product layer, even after exposure to SO_2_ gas for several hours, a considerable amount of CaO in the core area still remained unreacted for coarse particles [14]. Therefore, it is commonly believed that smaller particles can achieve a faster reaction rate and a higher calcium conversion [15]. However, some researchers noted that under actual CFB conditions, the residence time of very fine particles was restricted and could not meet the requirement of the contact time for SO_2_ capture [16,17]. Thus, it was considered that the optimum sorbent particle size should be close to the circulating ash for a longer residence time and a relatively higher reaction rate. Nevertheless, recent industrial practices have found that fine and even ultrafine (<10 μm) limestones can realize a high SO_2_ capture efficiency with a low Ca/S molar ratio [2,18], so it is meaningful to investigate the optimum sorbent particle size under different conditions. Among all the influencing factors for the particle size optimization, the reactivity at low SO_2_ concentrations is most significant.

A brief summary of investigations of a broad range of particle sizes under atmospheric conditions is listed in Table 1. Although many researchers have investigated the effects of the limestone particle size on the reaction between SO_2_ and CaO, there are just a few research results of a certain type of limestone whose particle size ranges from several micrometers to several hundred micrometers. Combining the experimental data from different scholars can be a possible way to solve this issue, but the differences of the limestone properties and the calcination conditions may cause some deviation in model prediction [19,20]. Adánez et al. [21] compared three different structural sulfation models and found that the same model parameters could not predict the conversion curves of different particle sizes of sorbents. Modification of model parameters with respect to the limestone particle size should be introduced for better prediction. Based on the ideas of the shrinking core model, Obras-Loscertales et al. [22] proposed a two-step sulfation model, which can predict the sulfation conversion of particles between 200 μm and 630 μm with similar parameters. However, the fitting thickness of the product layer, which was about 30 μm, could not be suitable for the particles smaller than 60 μm, otherwise the product layer will be thicker than the sorbent particle. To establish the sulfation model which is valid for different particle sizes, it is very important to first obtain the sulfation conversion curves covering the range of particle sizes from several micrometers to several hundred micrometers.

Additionally, both the reaction rate and the final sulfation conversion will strongly be affected by SO_2_ concentration. Most experiments are undertaken in a relatively high SO_2_ concentration over 1000 ppm, but the SO_2_ emission standards for CFB boilers in most countries are normally lower than 100 ppm [23]. What is worse, with the implement of the updated national emission regulation in China, SO_2_ emission is required to be not higher than 35 mg/Nm^3^ (~12 ppm) [24,25]. Most models regard the CaO sulfation as a first-order reaction [19,26], but Borgwardt et al. [20] obtained the apparent reaction orders of 0.62 ± 0.07. To obtain a better prediction at low SO_2_ concentrations, it is worthy to investigate the desulfurization characteristics at different SO_2_ concentrations, especially at the low SO_2_ concentrations.

Thermogravimetric analyzers (TGAs), fixed bed reactors and bubbling bed reactors are commonly used to study the effects of particle size on sulfation reaction. Although the heat and mass transfer conditions in a bubbling bed reactor are rather similar to that in a CFB boiler, it is not suitable for fine particles because of the particle escape. A fixed bed reactor can easily overcome this issue, but SO_2_ concentrations at the reactor outlet are always lower than the main stream, so differential conditions cannot be achieved at the beginning of the reaction. In addition, measurement of the gas component is also limited by the response and accuracy of the instrument. The TGA has satisfactory repeatability and accuracy, but small crucibles will lead to the particle packing, which will restrict the reaction due to the external mass transfer.

Therefore, in this paper, a large-capacity TGA (LC-TGA) was developed to investigate the effects of limestone particle size on the sulfation reactivity at low SO_2_ concentrations. Furthermore, an empirical model was proposed based on the experimental data.

## 2. Materials and Methods

**Figure 1:**
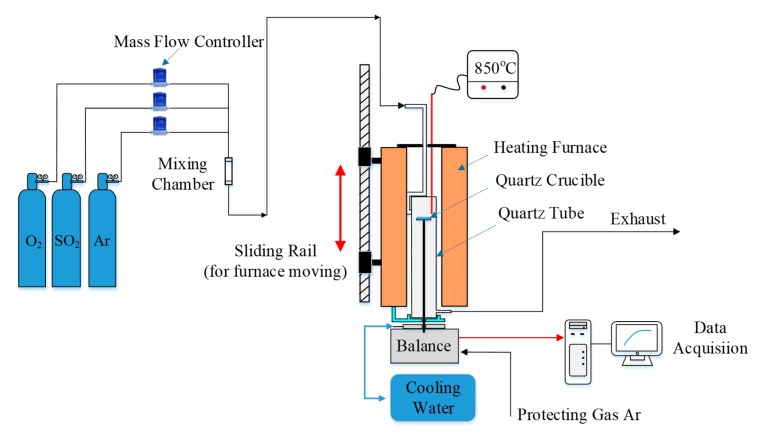
The schematic of the large-capacity thermogravimetric analyzer (LC-TGA) system.

The schematics of the LC-TGA is shown in Figure 1. Gaseous mixture through the mixture chamber was introduced from the top of the quartz tube to react with limestone samples on a quartz crucible (Shengfan Shiying Corporation, Lianyungang City, Jiangsu Province, China), whose diameter was 32 mm. The quartz tube was fixed with the heating furnace (Yuzhi Mechanical and Electrical Corporation, Shanghai, China), which can slide along the rail at the highest velocity of 10 mm/s. A K-type thermocouple, whose measurement accuracy is ±0.4%, was placed above the quartz crucible to record the reaction temperature. Mass variation of limestone samples was automatically recorded by a MT-WKX204 analytical balance produced by Mettler Toledo (Zurich, Switzerland). The maximum weight is over 100 g, and the readability can be down to 0.1 mg. The analytical balance was installed inside a water-cooled jacket, which was fixed on the ground to ensure stable readings. Inert Ar atmosphere was introduced into the water-cooled jacket to create a positive pressure environment, so the balance can be protected from the high temperature corrosion problems. A rubber seal O ring was installed between the quartz tube and the water-cooled jacket to prevent gas leakage, thus the exhaust gas was the mixture of the reaction gas and the protecting gas.

**Figure 2:**
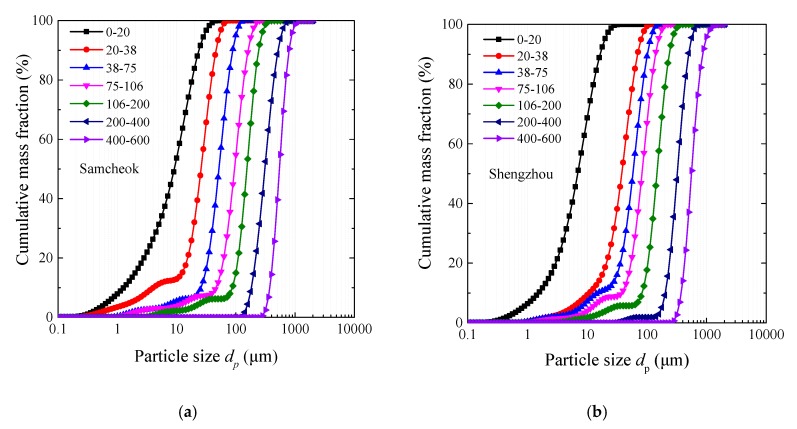
The particle size distributions of two limestone samples with narrow cut. (**a**) Korea Samcheok; (**b**) China Shengzhou.

Two types of limestone samples from China and Korea were sieved by an ultrasonic sieving machine (Sanyuantang Mechanical Corporation, Xinxiang County, Henan Province, China) into seven groups with narrow cuts, including 0–20 μm, 20–38 μm, 38–75 μm, 75–106 μm, 106–200 μm, 200–400 μm and 400–600 μm. The particle size distributions measured by Malvern are shown in Figure 2. The main cut sizes of the seven groups are listed in Table 2. The X-Ray Fluorescence (XRF) analysis for each size cut of limestone was performed, and the relative deviation of CaO content was smaller than 2%. Thus, the component was assumed the same for each type of limestone, and the average values are listed in Table 3.

**Figure 3:**
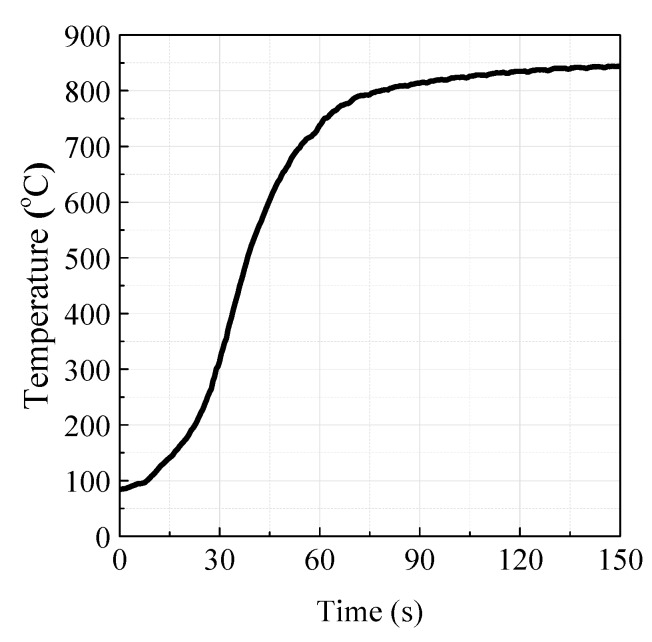
The heating curve of crucible in LC-TGA (5 standard liters per minute at 1 atm, 0 °C (SLM)).

Before the experiment, 10–30 mg of limestone samples was uniformly dispersed on the crucible with deionized water, and then dried below 150 °C in an oven. At the beginning, the furnace was lifted to the highest height and heated to 850 °C with Ar flux through the quartz tube. After the crucible was installed and the readings of the analytical balance was stable, the heating furnace was moved downward at a speed of 5 mm/s. As shown in Figure 3, the maximum increasing rate of temperature could reach 15–20 K/s, which is much faster than that in most traditional commercial TGA, so the calcination condition in the LC-TGA is much closer to the injection condition in fluidized beds.

Limestone samples were calcined under Ar atmosphere for 5 min and then the calcium oxide reacted with sulfur containing gas for 20 min. At the initial stage of the experiment, the mass of the limestone sample decreased quickly due to calcination. The calcination was assumed to finish after the sample mass was stable. After Ar was switched to the gas mixture of Ar, O_2_ and SO_2_, the sulfation reaction occurred and the sample mass increased. The O_2_ concentration in the LC-TGA experiment remained unchanged at 3.5%, which is similar to that in CFB boilers. The blank experiment under the same heating rate and gas atmosphere was conducted for each set of experiments to eliminate the effects of gas flow and buoyancy on the mass measurement. With the assumption that the impurities in the sample remained constant in the reactions, the limestone conversion can be calculated by the following Equations (1)–(3):

(1) $$n_{CaO}=\frac{m_{1}\cdot LOF\cdot \gamma }{M_{CaO}}$$

(2) $$n_{CaSO_{4}}\left(t\right)=\frac{m_{3}\left(t\right)-m_{2}}{M_{SO_{3}}}$$

(3) $$X_{s}\left(t\right)=\frac{n_{CaSO_{4}}\left(t\right)}{n_{CaO}}$$

where $n_{CaO}$ is the mole number of CaO after calcination, mol; $n_{CaSO_{4}}$ is the mole number of CaSO_4_ at a given time *t*, mol; $M_{SO_{3}}$ is the molecular mass of SO_3_, g/mol; *m*_1_ and *m*_2_ are the limestone mass before and after the calcination respectively, g; and *m*_3_(*t*) is the sample mass at a given time *t* during the desulphurization reaction, g.

The measurement results of LC-TGA was also compared with that in a fixed bed reactor (Shengfan Shiying Corporation, Lianyungang City, Jiangsu Province, China), which had the same gas controlling system. The inner diameter of the fixed bed reactor was 18 mm. Silica wool was compacted and spread on the quartz sintered distributor, preventing fine particles carried by gas flow blocking the quartz sintered distributor. Limestone samples (80 mg) were mixed well with 1.5 g quartz sands with a Saunter diameter of 150 μm, and then uniformly spread on the silica wool. During the heating process, pure CO_2_ was introduced into the fixed bed reactor to inhibit limestone from decomposition, which was also adopted by previous studies [19]. After the furnace was heated to the given temperature (850 °C), pure CO_2_ was switched to Ar to start limestone calcination. After 5–10 min of limestone calcination, Ar was switched to gas mixture of Ar and SO_2_ to start CaO sulfation. The total gas flow rate was set as 2 SLM (standard liter per minute at 1 atm, 0 °C). CO_2_ and SO_2_ concentrations were measured by a mass spectrum analyzer with a frequency of 0.75 Hz. The sulfation conversion can be obtained by the following Equations (4) and (5):

(4) $$n_{CaSO_{4}}\left(t\right)=\Delta n_{SO_{2}}=\frac{\left(P_{SO_{2},0}-P_{SO_{2}}\left(t\right)\right)V}{RT_{A}}=\frac{P_{A}V_{A}}{RT_{A}}\int_{t_{1}}^{t}\left(C_{0,SO_{2}}-C_{SO_{2}}\left(t\right)\right)dt$$

(5) $$X_{s}\left(t\right)=\frac{n_{CaSO_{4}}\left(t\right)}{n_{CaO}}$$

where $P_{{\mathrm{SO}}_{2}}$ is the partial pressure of SO_2_, Pa; *P_A_* is the atmospheric pressure, 1 atm; *V_A_* is the total gas flow rate under the standard condition (273.15 K, 1 atm), m^3^/s; *R* is the ideal gas constant, J/(mol·K); *T_A_* is the atmospheric temperature, 273.15 K; $C_{{\mathrm{SO}}_{2}}\left(t\right)$ is the outlet SO_2_ concentration at time *t*, mol/mol; $C_{0,{\mathrm{SO}}_{2}}$ is the inlet SO_2_ concentration, mol/mol.

In addition, a commercial TGA-Q500 produced by TA Instruments (New Castle, DE, USA) was also used to validate the measurements of the LC-TGA. The TGA-Q500 had a maximum heating rate of 50 K/min and a maximum gas volume flow rate of 200 mL/min. Limestone samples (2 mg) were used in each experiment with a gas volume flow rate of 100 mL/min. The experimental procedure for the TGA-Q500 was approximately the same as that for the LC-TGA except that the limestone was calcined at a given heating rate instead of a given environmental temperature. Two heating rates of 10 K/min and 30 K/min were both studied in the experiments.

## 3. Results and Discussion

### 3.1. Test of the LC-TGA

**Figure 4:**
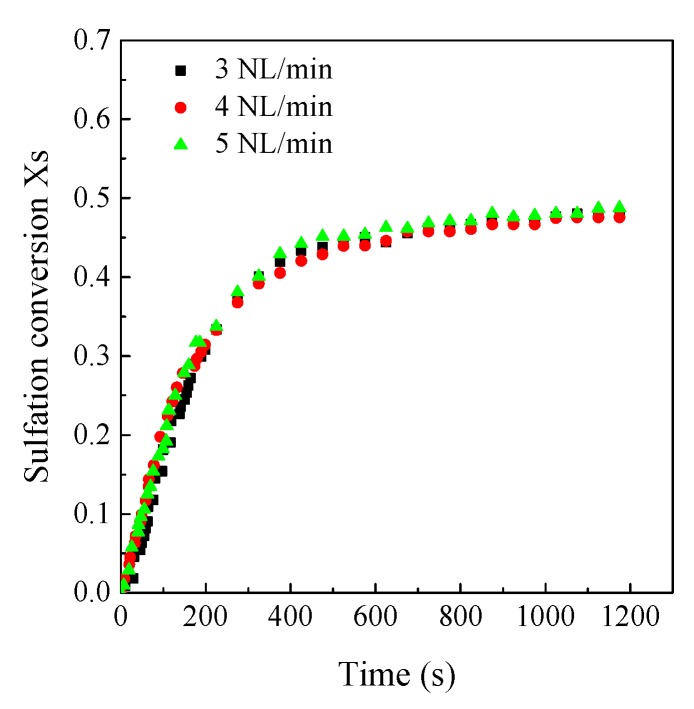
The sulfation conversion under different gas flow rate (Shengzhou limestone, 3.5 μm, 850 °C, 500 ppm).

**Figure 5:**
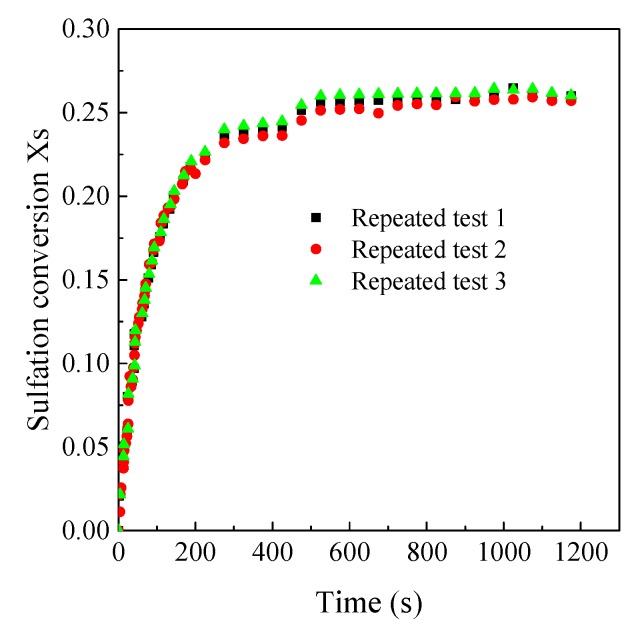
The repeated test results (Samcheok limestone, 26.3 μm, 850 °C, 1000 ppm).

As shown in Figure 4, in order to eliminate the effects of external mass transfer on the sulfation reaction, experiments under different gas flow rates were compared. The sulfation conversion remained almost unchanged when the gas mixture was higher than 3 SLM. Thus, the volume flow rate of gas mixture was set to be 5 SLM in the experiments, which can eliminate the effects of external mass transfer on the sulfation reaction at different conditions. Figure 5 shows the reproducibility of the LC-TGA. It can be seen that the reproducibility is sufficient despite some data fluctuation which is lower than 0.2 mg, thus the estimated measurement data error is below ±4%.

### 3.2. Comparison of Sulfation Conversion in Different Reactors

**Figure 6:**
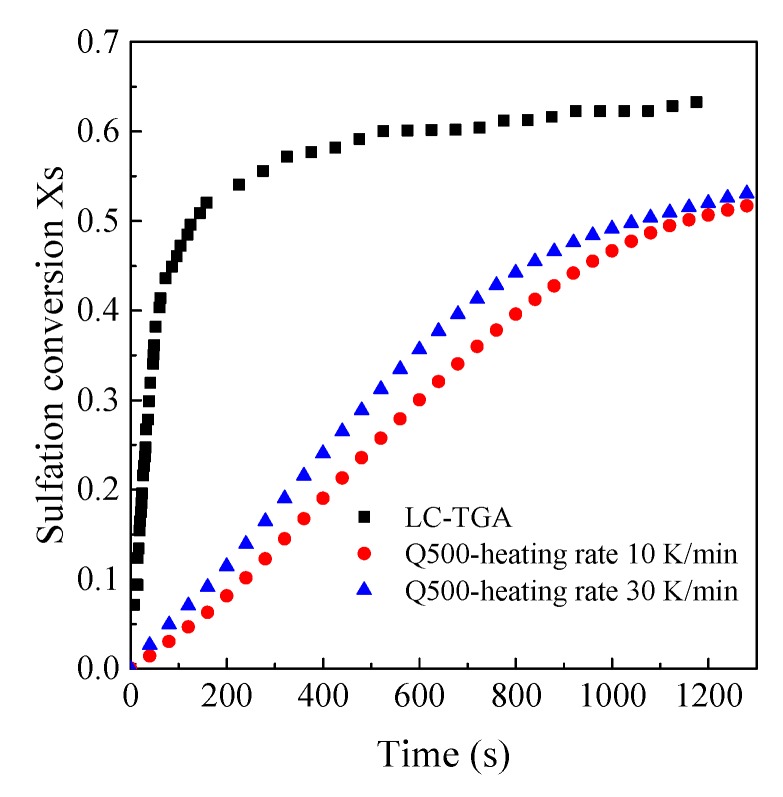
The comparison of sulfation conversion in the LC-TGA and the TGA-Q500 (Shengzhou limestone, 3.5 μm, 850 °C, 2000 ppm).

The comparison of sulfation conversion in the LC-TGA and the TGA-Q500 is illustrated in Figure 6. The sulfation conversion in the LC-TGA was apparently much faster than that in TGA-Q500, although the conversion increased with the heating rate in the TGA-Q500. The time to reach a conversion of 0.5 in the TGA-Q500 was almost ten times higher than that in the LC-TGA. The high chemical reaction rate is mainly ascribed to three factors. First, limestone samples can be dispersed more uniformly in the LC-TGA with a larger crucible than that in the TGA-Q500. Particle packing can be alleviated especially for the fine powders thus the external mass transfer was improved. Second, a larger specific surface area can be obtained at a higher calcination rate in the LC-TGA, thus the porous structure promoted the sulfation reactivity. Lastly, the maximum reaction gas flow rate seemed insufficient in these cases for eliminating the external mass transfer in the TGA-Q500 due to the instrument limit, so the conversion rate was also influenced by the gas flow rate.

**Figure 7:**
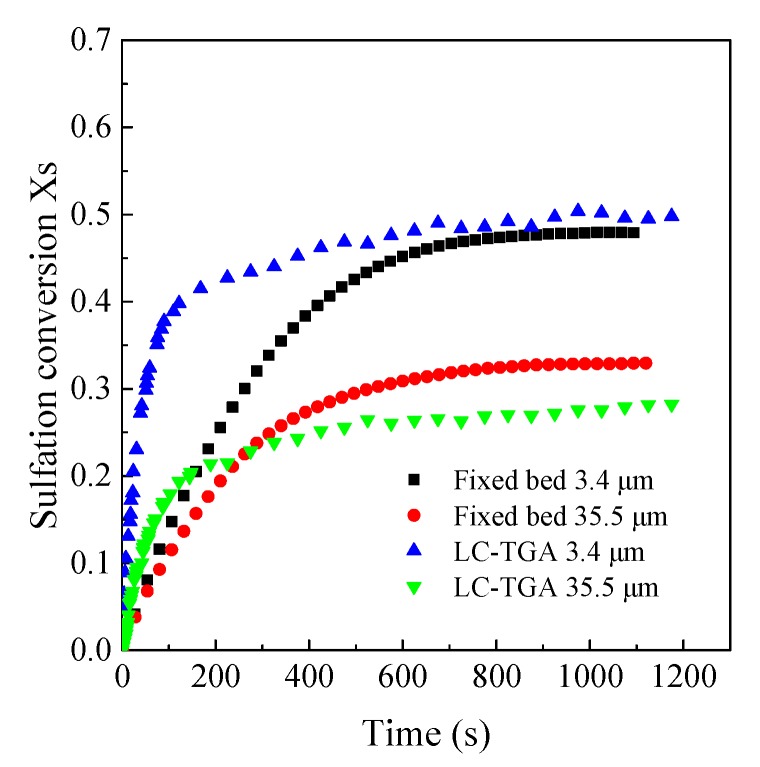
The comparison of sulfation conversion in the LC-TGA and the fixed bed reactor (Samcheok limestone, 850 °C, 2000 ppm).

As shown in Figure 7, the sulfation conversions increased rather fast at the first beginning of reaction, and then turned into a slow increase both in the LC-TGA and the fixed bed reactor. However, the calcium conversion rates at the initial stage of the reactions in the LC-TGA were much faster than those in the fixed bed reactor. It is commonly known that the sulfation process is performed in two stages. The first one is fast and controlled by the chemical reaction and gas diffusion through the pore structure of particles. The second one is slower and controlled by the ion diffusion through the CaSO_4_ production layer [22]. As the outlet SO_2_ concentrations in the fixed bed reactor sharply decreased to nearly zero at the beginning of the reaction during experiments, the differential operating conditions could not be achieved. Thus, the initial CaO conversion rate in the fixed bed reactor was significantly deviated from the intrinsic reaction rate, which was limited by the external gas diffusion. In contrast, less sample mass and higher gas flow rates could be used in the LC-TGA, thus the SO_2_ concentration around the CaO particles was much closer to that in the main stream, leading to a higher initial conversion rate in the LC-TGA. After the reaction goes to the second stage, the major control mechanism changes to the diffusion through the production layer, so conversion rates in both reactor systems became much closer.

Based on the above discussion, it can be concluded that the LC-TGA can achieve a faster calcination rate, and greatly alleviate the negative effects of the external mass transfer on the measurements of CaO sulfation reactivity, showing a good reliability for measuring the sulfation reaction with different particle sizes.

### 3.3. Effects of Limestone Particle Size on the CaO Sulfation Reactivity

**Figure 8:**
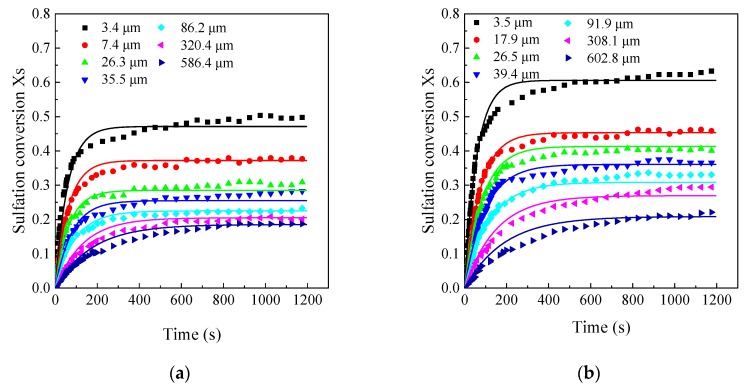
Effects of limestone particle size on the CaO sulfation reactivity (the large capacity TGA, 850 °C, 2000 ppm): (**a**) Samcheok; (**b**) Shengzhou.

Sulfation conversion rate with different particle sizes in the LC-TGA are shown in Figure 8. It is also observed that the final CaO conversion of the finest particle was significantly higher than that of other particle sizes. Particle size has crucial effects on the final calcium conversion. As mentioned above, the sulfation reaction blocks the surface pores, leading to an unreacted inner core. Thus, when the particle size decreased, the final CaO conversion also increased. Thus, the calcium utilization rate was improved greatly when the limestone particle size reduced to less than 20 μm or even 10 μm. In addition, the chemical reaction rates were also affected by limestone particle size. CaO conversion rates of the finer particles were higher than those of the coarser particles, especially at the very initial stage of the reactions. Thus, it can be concluded that finer limestone particles had a better CaO sulfation reactivity with higher chemical reaction rate and final conversion.

### 3.4. Effects of SO_2_ Concentration on the CaO Sulfation Reactivity

**Figure 9:**
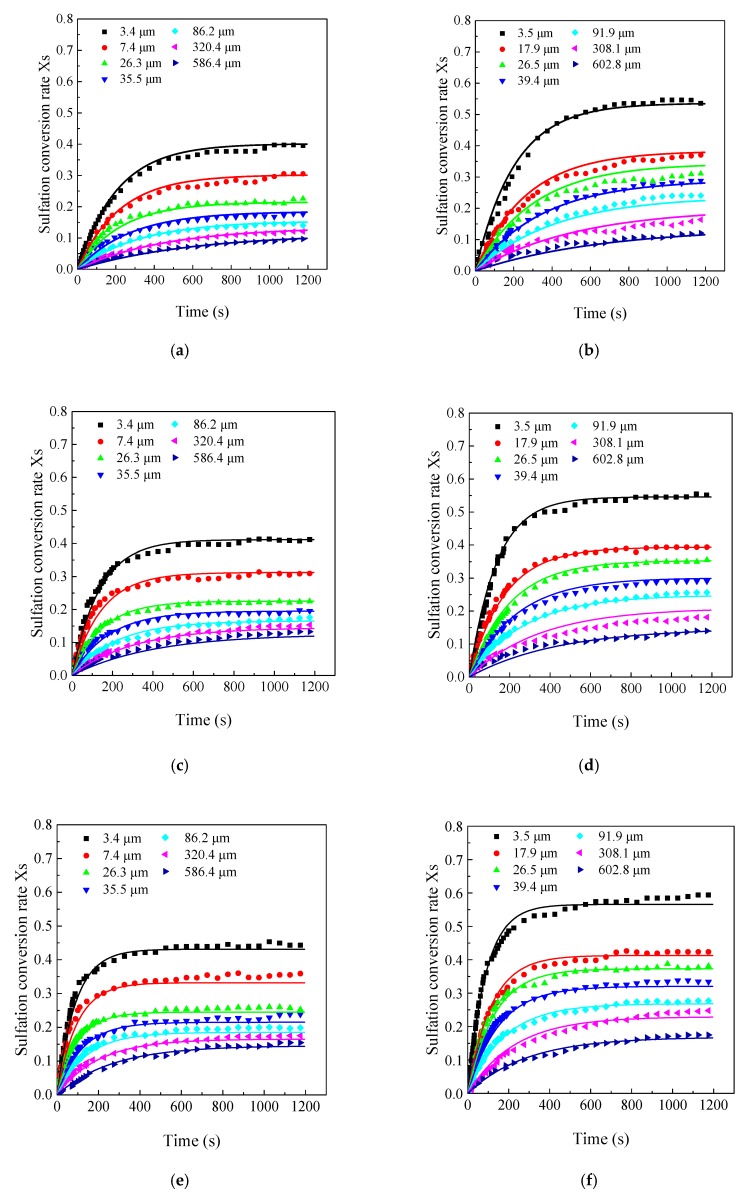
The sulfation conversion at different SO_2_ concentrations under 850 °C: (**a**) Samcheok, 250 ppm; (**b**) Shengzhou, 250 ppm; (**c**) Samcheok, 500 ppm; (**d**) Shengzhou, 500 ppm; (**e**) Samcheok, 1000 ppm; (**f**) Shengzhou, 1000 ppm.

**Figure 10:**
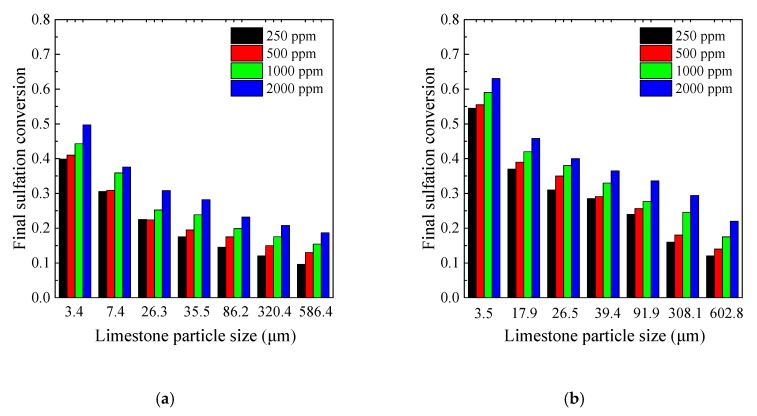
The final sulfation conversion at different SO_2_ concentrations under 850 °C: (**a**) Samcheok; (**b**) Shengzhou.

Figure 9 and Figure 10 show the sulfation process at different SO_2_ concentrations, and both the final sulfation conversion and the chemical reaction rate decreased with the SO_2_ concentrations. At 250 ppm SO_2_, the final CaO conversion of 600 μm particles was only 10% while the final CaO conversion of 3.4 μm particles was still as high as 40% for Samcheok limestone. The reduction of the final sulfation conversion from 2000 ppm to 250 ppm was similar for different particle sizes, thus low final sulfation conversion of the coarser limestone restricted it from realizing ultra-low SO_2_ emission in CFB boilers even with the long residence time. In contrast, ultrafine limestone particles still showed a good sulfation reactivity even at low SO_2_ concentrations. If the contact time can be ensured, it is more likely to realize ultra-low SO_2_ emission at low Ca/S ratios by application of finer limestone.

**Figure 11:**
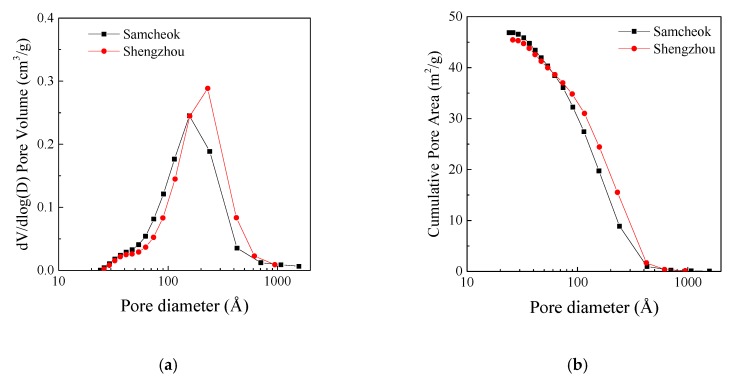
Pore size distribution of CaO after the calcination in the LC-TGA: (**a**) Barrett-Joyner-Halenda (BJH) adsorption dV/dlog(D) (V is the pore volume, and D is the pore size) pore volume; (**b**) BJH adsorption cumulative pore area.

In addition, Shengzhou limestone showed a better reactivity than Samcheok limestone, both in final conversion and reaction rate. The pore size distributions of these two kinds of limestone were measured by nitrogen adsorption apparatus ASAP 2460 produced by Micromeritics Instruments Corporation (Norcross, GA, USA), as shown in Figure 11. The measured BET (Brunauer–Emmett–Teller) specific surface areas of Shengzhou and Samcheok limestones were 38.07 m^2^/g and 39.23 m^2^/g, respectively, and the BJH (Barrett-Joyner-Halenda) adsorption cumulative pore volume were 0.177 cm^3^/g and 0.156 cm^3^/g, respectively. Although CaO particles calcined from these two kinds of limestone had similar pore surface area, the mean pore size of Shengzhou CaO was larger than that of Samcheok CaO. Previous studies have found that smaller pores will be more easily plugged and lead to the premature termination of sulfation [32]. Thus, a better pore structure of Shengzhou CaO may have enhanced its sulfation reactivity. The detailed analysis still needs further studies in the future.

### 3.5. Model Prediction

Many researchers have developed sulfation models to predict the conversion rate under different conditions and coupled them with gas-solid flow models to calculate desulfurization efficiencies in industrial CFB boilers. Rubiera et al. [33] proposed a classic semi-empirical model, which was widely adopted in CFB models. Two empirical parameters of *X*_s,max_ and *K_c_*^0^ are used to predict CaO sulfation reactions as the following equation,

(6) $$X_{s}=X_{s,\max}(1-\exp(-\frac{K_{c}{}^{0}C_{{\mathrm{SO}}_{2}}t}{X_{s,\max}}))$$

where *X*_s,max_ is the maximum sulfation conversion after infinite reaction time; *K_c_*^0^ is the apparent reaction rate constant at the initial reaction, m^3^/(mol·s); *C*_SO_2__ is the SO_2_ concentration at the particle surface, kmol/m^3^. Thus, the conversion rate at a given time *t* can be calculated as the following equation,

(7) $$\frac{dX_{s}}{dt}=K_{c}{}^{0}C_{{\mathrm{SO}}_{2}}\exp(-\frac{K_{c}{}^{0}C_{{\mathrm{SO}}_{2}}t}{X_{s,\max}})$$

**Figure 12:**
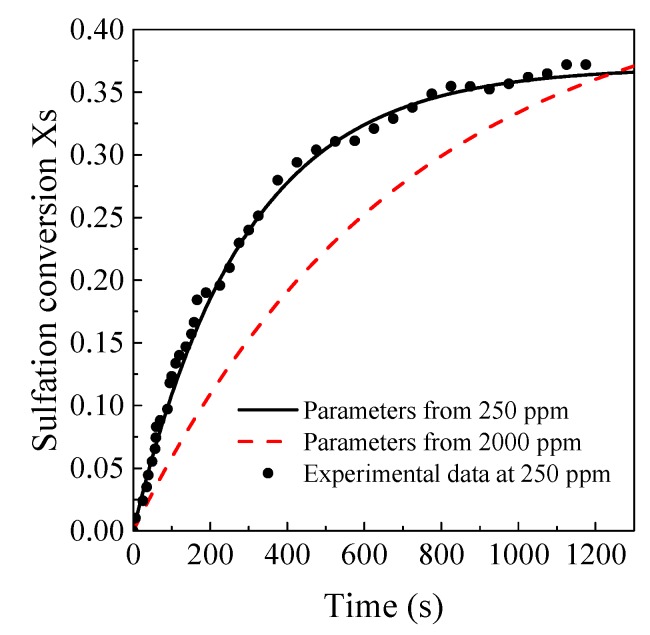
Predicted sulfation conversion at 250 ppm using empirical parameters from different SO_2_ concentration using Equation (6) (Shengzhou Limestone, 17.9 μm).

Using this model to fit with the experimental results, *X*_s,max_ and *K_c_*^0^ of the two kinds of limestone at different SO_2_ concentrations were obtained and are listed in Table 4. *X*_s,max_ and *K_c_*^0^ decreased significantly with the increase in particle size, which is in agreement with previous studies. Besides, it was also found that *X*_s,max_ and *K_c_*^0^ were affected by SO_2_ concentrations. When SO_2_ concentrations increased from 250 ppm to 2000 ppm, *K_c_*^0^ gradually decreased and *X*_s,max_ increased. *K_c_*^0^/*X*_s,max_ was double at 250 ppm than that at 2000 ppm. Thus, if the model parameters obtained from the experimental results at high SO_2_ concentrations are used to predict sulfation process at low SO_2_ concentrations, the sulfation reaction rate may be underestimated, leading to an overestimating outlet SO_2_ concentration. The residence time of limestone particles with a similar size as the circulating ash was sufficiently long, so the bias of this model may not be obvious at high SO_2_ concentrations. However, as shown in Figure 12, the deviation will be much more severe at the low SO_2_ concentration for finer limestone particles. As the residence time for the fine limestones were restricted, this model may not be satisfactory in predicting the low SO_2_ emission at the boiler outlet.

The modeling bias at low SO_2_ concentrations means that the apparent reaction order with respect to $C_{{\mathrm{SO}}_{2}}$ should be lower than 1. Thus, in order to predict CaO sulfation with a broad range of particle sizes at low SO_2_ concentrations, a modified empirical model was proposed in this paper as the following expression:

(8) $$X_{s}=X_{s,\max}(C_{{\mathrm{SO}}_{2}},d_{p})(1-\exp(-K_{c}(d_{p})C_{{\mathrm{SO}}_{2}}^{m}t))$$

where *X*_s,max_(*C*_SO_2__,*d_p_*) is the final CaO conversion with a given particle diameter *d_p_* at a given SO_2_ concentration *C*_SO_2__; *K_c_*(*d_p_*) is the apparent reaction rate constant with a given particle diameter *d_p_*, m^3−m^/(mol^1−m^·s); *m* is the apparent reaction order with respect to *C*_SO_2__.

**Figure 13:**
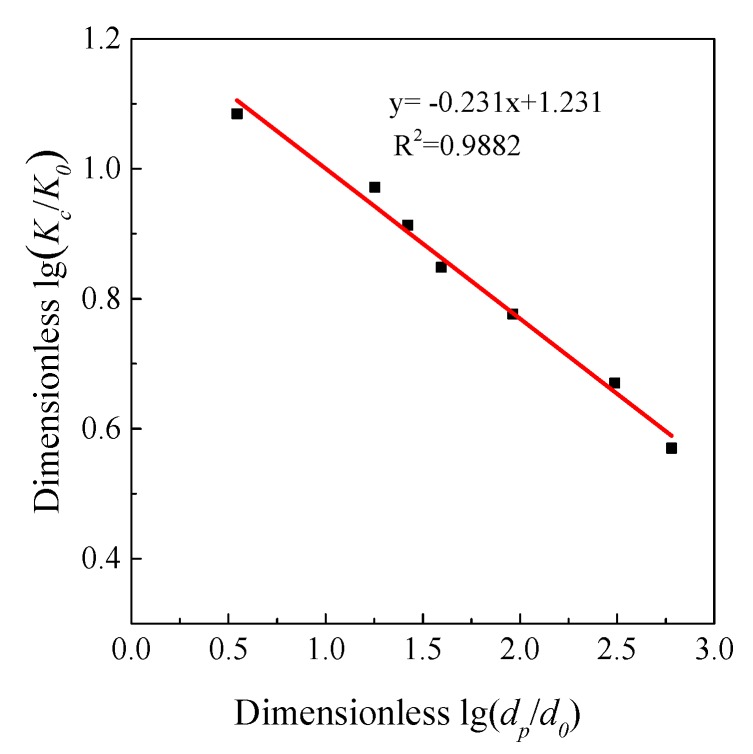
The relationship between apparent reaction rate constant (*K_c_*) and particle diameter (*d_p_*) of Shengzhou limestone.

**Figure 14:**
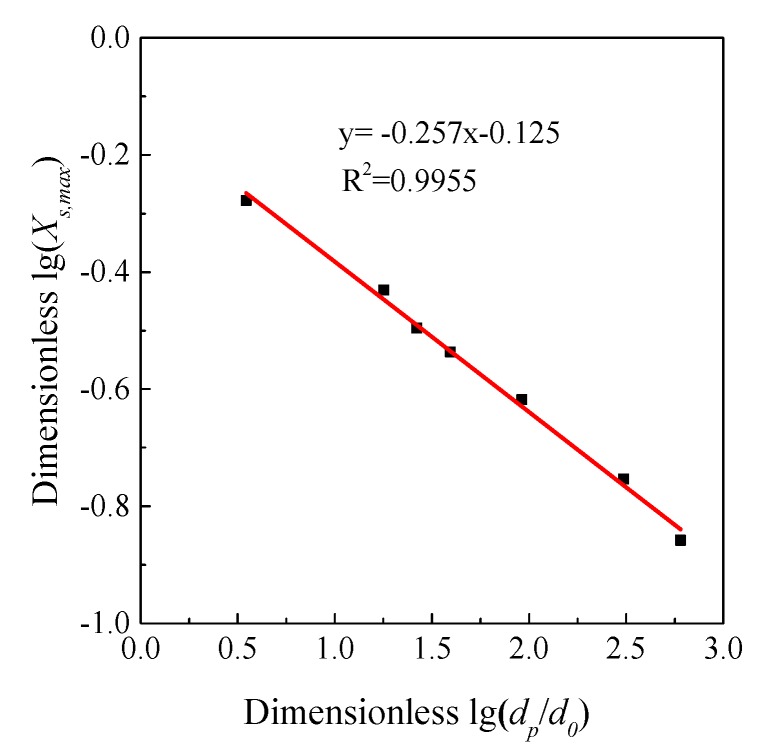
The relationship between *X*_s,max_ and *d_p_* of Shengzhou limestone (500 ppm SO_2_).

The apparent reaction orders with respect to *C*_SO_2__ of Shengzhou limestone and Korea limestone were 0.60 and 0.61 using optimal linear fitting. Borgward et al. [20] also supposed that the reaction order *m* with respect to $C_{{\mathrm{SO}}_{2}}$ should be 0.62 ± 0.07 and will be affected by limestone types. Therefore, the modification of the apparent reaction order was reasonable. As shown in Figure 13 and Figure 14, *K_c_* and *X*_s,max_ for each particle size of Shengzhou limestone are plotted logarithmically against particle size *d*_32_, and strong negative correlations can be obviously seen.

The fitting reaction models of Shengzhou and Samcheok limestones are described as the following two equations, respectively.

(9) $$X_{s}=\left(0.020\times \frac{C_{so_{2}}-C_{so_{2},0}}{C_{so_{2},0}}+0.7496\cdot {\left(\frac{d_{p}}{d_{0}}\right)}^{-0.257}\right)\left(1-exp(-17.036\cdot {\left(\frac{d_{p}}{d_{0}}\right)}^{-0.231}\cdot C_{{\mathrm{SO}}_{2}}^{0.61}t\right))$$

(10) $$X_{s}=\left(0.020\times \frac{C_{so_{2}}-C_{so_{2},0}}{C_{so_{2},0}}+0.4834\cdot {\left(\frac{d_{p}}{d_{0}}\right)}^{-0.221}\right)\left(1-exp(-15.789\cdot {\left(\frac{d_{p}}{d_{0}}\right)}^{-0.210}\cdot C_{{\mathrm{SO}}_{2}}^{0.60}t\right))$$

where *C*_SO_2___,0_ = 5.4262 × 10^−6^ kmol/m^3^ (500 ppm); *d_p_* is limestone particle diameter, μm; *d*_0_ is the characteristic particle size, 1 μm.

The comparison between the experimental results and the modeling predictions are shown in Figure 8 and Figure 9. At the initial reaction stage, the modeling predictions agreed well with the experimental results. While at the second reaction stage, there was a little bias between the modeling predictions and the experimental results. The modeling predictions showed that CaO conversion gradually approached the maximum conversion after the initial quick reaction stage. However, according to the product-layer diffusion theory, CaO still reacts with SO_2_ slowly and the conversion will also increase slowly after the initial quick reaction stage. This may be the main reason for the bias of the model predictions in the later reaction stage. However, this bias seems acceptable because the increase in sulfation conversion at low SO_2_ concentration was subordinate to the increase in the initial stage. Thus, it can be assumed that this empirical model can predict CaO sulfation with different particle sizes at different SO_2_ concentrations, especially at low SO_2_ concentrations. In addition, this model form is very simple to incorporate into available FBC models to predict SO_2_ emissions for industrial applications. When using this empirical model, if it is not allowed to thoroughly study an unknown limestone in the future, it is recommended to use m = 0.6 and measure at least three characteristic particle sizes at a typical SO_2_ concentration, and then the limestone reactivity can be approximately determined.

## 4. Conclusions

A large-capacity TGA was developed in this paper to investigate the effects of limestone particle size on the sulfation reactivity at low SO_2_ concentrations, which showed a better measurement performance of the sulfation conversion than a commercial TGA-Q500 and a fixed bed reactor, especially at the initial stage of fast reaction. The experimental results showed that finer limestone particles have a better reactivity in the final conversion and faster chemical reaction rate. With the decrease of SO_2_ concentration, both the final calcium conversion and the sulfation conversion rate decreased, but the ultrafine limestone particles still showed a good sulfation reactivity even at 250 ppm SO_2_. If the residence time can be ensured, it is more likely for ultra-fine limestone to realize ultra-low SO_2_ emissions at low Ca/S ratios.

An empirical sulfation model was established based on the experimental results. Both the final conversion and the apparent reaction rate constant had strong negative correlations against particle size, and the fitting reaction order of SO_2_ was found to be about 0.6, which can well predict the sulfation process of different limestone particle sizes at low SO_2_ concentrations. The model form is very simple to incorporate into available FBC models to predict SO_2_ emissions.

## Figures and Tables

**Table 1:** A brief summary of investigations of a broad range of particle sizes under atmospheric conditions.

Author	Method	Particle Size (μm)	Temperature (°C)	Atmosphere	SO_2_ Concentration (ppm)
Simons [27]	Fixed Bed Reactor	1–78	850	N_2_ + O_2_ + CO_2_ + H_2_O + SO_2_	297–315
Zarkanitis [28]	TGA	53–350	700–850	N_2_ + O_2_ + SO_2_	3000–5000
Milne [29]	Dispersed-Phase Isothermal Reactor	4.1–49	980–1171	N_2_ + O_2_ + SO_2_	1480
Adánez [21,26]	TGA Bubbling Bed Reactor	158–1788	800–900	N_2_ + O_2_ + SO_2_ + CO_2_	2500
Mattisson [19]	Fixed Bed Reactor	45–2000	850	N_2_ + O_2_ + SO_2_ + CO_2_	1500
Fan [15]	Differential Bed Reactor	7.5–150 (modified)	900	N_2_ + O_2_ + SO_2_	3900
Laursen [30]	Fixed Bed	212–355	850	N_2_ + O_2_ + SO_2_	2250
Abanades [31]	TGA	70–1000	850	N_2_ + O_2_ + SO_2_	500–5000
Obras-Loscertales [22]	TGA Bubbling Bed Reactor	200–630	800–950	N_2_ + O_2_ + SO_2_ + CO_2_	1500–4500

**Table 2:** The main cut sizes of the seven groups of the two kinds of limestone samples.

	Korea Samcheok				China Shengzhou			
Particle Size (μm)	*d*_10_	*d*_50_	*d*_90_	*d*_32_	*d*_10_	*d*_50_	*d*_90_	*d*_32_
0–23	1.4	10.3	26.7	3.4	1.7	7.8	18.4	3.5
20–38	4.5	29.1	51.9	7.4	10.8	37.9	69.7	17.9
38–75	29.1	58.2	99.3	26.3	16.7	65.4	114.0	26.5
75–106	52.0	106.5	180.7	35.5	42.8	93.6	158.0	39.4
106–200	98.2	172.6	271.3	86.2	93.5	165.4	265.1	91.9
200–400	205.8	346.2	577.9	320.4	228.1	362.4	560.0	308.1
400–600	421.4	609.7	886.1	586.4	421.9	627.2	963.3	602.8

**Table 3:** The X-Ray Fluorescence (XRF) component analysis of the two kinds of limestone samples.

Parameters	LOI *	CaO	MgO	SiO_2_	Al_2_O_3_	Na_2_O	Fe_2_O_3_	Others
Korea Samcheok	42.40	52.82	2.42	0.92	0.58	0.16	0.37	0.32
China Shengzhou	42.75	55.92	0.21	0.38	0.20	0.16	0.06	0.21

* Loss on ignition.

**Table 4:** Maximum sulfation conversion after infinite reaction time (*X*_s,max_) and the apparent reaction rate constant at the initial reaction (*K_c_*^0^) of the two kinds of limestones at different SO_2_ concentrations.

**Shengzhou Limestone**									
Particle size (μm)	*d*_50_		7.8	37.9	65.4	93.6	165.4	362.4	627.2
	*d*_32_		3.5	17.9	26.5	39.4	91.9	308.1	602.8
SO_2_ concentration (ppm)	250	*K_c_* ^0^	805.4	473.4	385.7	298.0	234.0	126.9	90.3
		*X*_s,max_	0.53	0.37	0.32	0.29	0.24	0.18	0.14
	500	*K_c_* ^0^	728.6	384.0	291.9	232.8	168.3	94.2	71.2
		*X*_s,max_	0.54	0.39	0.34	0.30	0.25	0.21	0.15
	1000	*K_c_* ^0^	664.1	292.4	255.6	203.4	145.4	87.9	48.3
		*X*_s,max_	0.59	0.41	0.36	0.32	0.27	0.24	0.19
	2000	*K_c_* ^0^	563.4	230.7	221.1	163.7	118.2	82.8	30.6
		*X*_s,max_	0.62	0.44	0.39	0.37	0.32	0.27	0.22
**Samchoek Limestone**									
Particle size (μm)	*d*_50_		10.3	29.1	58.2	106.5	172.6	346.2	609.7
	*d*_32_		3.4	7.4	26.3	35.5	86.2	320.4	586.4
SO_2_ concentration (ppm)	250	*K_c_* ^0^	732.6	487.4	381.2	251.4	184.4	117.1	90.1
		*X*_s,max_	0.39	0.29	0.21	0.17	0.15	0.14	0.11
	500	*K_c_* ^0^	673.9	415.4	311.0	213.0	145.8	100.5	58.7
		*X*_s,max_	0.40	0.30	0.22	0.18	0.16	0.15	0.13
	1000	*K_c_* ^0^	560.4	327.5	290.1	205.4	130.7	87.9	41.3
		*X*_s,max_	0.42	0.32	0.26	0.21	0.18	0.17	0.15
	2000	*K_c_* ^0^	437.9	262.8	177.2	166.4	108.6	66.4	36.3
		*X*_s,max_	0.45	0.35	0.29	0.24	0.22	0.19	0.18
